# Hypercysteinemia, A Potential Risk Factor for Central Obesity and Related Disorders in Azores, Portugal

**DOI:** 10.1155/2019/1826780

**Published:** 2019-06-20

**Authors:** Ana Lima, Rita Ferin, Mafalda Bourbon, José Baptista, M. Leonor Pavão

**Affiliations:** ^1^DCFQE/Faculty of Sciences and Technology, University of the Azores, 9501-855 Ponta Delgada, Portugal; ^2^Unidade de I&D, Grupo de Investigação Cardiovascular, Departamento de Promoção da Saúde e Prevenção de Doenças Não Transmissíveis, Instituto Nacional de Saúde Doutor Ricardo Jorge, Lisboa, Portugal; ^3^BioISI—Biosystems & Integrative Sciences Institute, Faculdade de Ciências, Universidade de Lisboa, Lisboa, Portugal

## Abstract

In Azores, the standardized mortality rate for coronary artery disease (CAD) is nearly the double when compared to mainland Portugal. The aim of this study was to compare the prevalence of conventional CAD risk factors, as well as the plasma aminothiol profile (and its major determinants), between two groups of healthy subjects from Ponta Delgada (in Azores) and Lisbon (in mainland) cities, searching for precocious biomarker(s) of the disease. The study groups consisted of 101 healthy volunteers from Ponta Delgada (PDL) and 121 from Lisbon, aged 20–69 years. No differences in the prevalence of classical CAD risk factors were found between the study groups, except in physical inactivity and related central obesity, which were both higher in PDL men than in those from Lisbon. Hypercysteinemia, which seems to result from sulfur-rich amino acid diets and/or vitamin B_12_ malabsorption, revealed to be significantly more prevalent in PDL vs. Lisbon subjects (18% vs. 4%, *P*=0.001), namely, in male gender. Moreover, plasma Cys levels predicted waist circumference (*β* coefficient = 0.102, *P*=0.032) and concomitant central obesity and were also associated with insulin resistance. Nevertheless, hyperhomocysteinemia prevalence was similar in both groups, despite the fact that PDL subjects exhibited a higher rate of vitamin B_12_ deficiency compared to those from Lisbon (19% vs. 6%, *P*=0.003). Owing to the nature of this study design, a cause-effect relationship between high plasma Cys levels and central obesity or CAD risk could not be derived, but results strongly suggest that hypercysteinemia is a potential risk factor for metabolic disorders, i.e., obesity and insulin resistance, and CAD in Azores, a hypothesis that asks for confirmation through further large prospective studies.

## 1. Introduction

Atherosclerotic cardiovascular diseases (CVD) are the main cause of death and disability in Portugal, where the Azores islands still have the highest standardized mortality rate for coronary artery disease (CAD), nearly twice the observed one in mainland Portugal [1].

Atherosclerosis is a chronic, inflammatory, multifactorial condition which can develop as a silent and progressive disease, whose underlying mechanisms are complex and can vary from one population to another [2]. Oxidative stress plays a key role in atherogenesis since the beginning and all along the progression of the pathology. It is defined as an imbalance between the production of reactive oxygen species (ROS) and their elimination by the antioxidant defense systems, with a prevailing pro-oxidant status that results in macromolecular damage and disruption of cellular redox signaling. Oxidative stress is the unifying mechanism for many CVD risk factors [3], such as obesity and diabetes mellitus, and it also activates proinflammatory signaling pathways. Since classical risk factors fail to account for part of the CVD cases and oxidative stress can have a causal role in these diseases [4], it is important to identify early, noninvasive oxidative stress biomarkers in healthy populations in order to define precociously subjects at high-risk for CVD.

Plasma aminothiols, such as homocysteine (Hcy), cysteine (Cys), cysteinylglycine (Cys-Gly), and glutathione (GSH), are actually seen as good markers for oxidative stress [5]. All these species interact via redox, namely, sulfide-disulfide exchange reactions, and their reduced, oxidized, and protein-bound forms comprise a dynamic system referred as the thiol redox status. Thiol redox status plays a central role in redox homeostasis, which is an essential condition for normal cellular functions [6]. However, when in excessive amounts, total plasma Hcy and Cys can have pro-oxidant effects, by producing ROS and concomitant endothelial dysfunction, as well as promoting LDL oxidation, thus favoring atherogenesis [7, 8]. High Cys-Gly concentration can also disrupt the thiol redox status in an iron-dependent manner [9]. On the other hand, GSH is the major intracellular nonprotein antioxidant [10].

Aminothiols are metabolically interrelated. Hcy is produced from methionine (Met), an essential amino acid, and can be remethylated to Met through the action of methionine synthase, which requires vitamin B_12_ as a cofactor and 5-methyltetrahydrofolate as a substrate. Alternatively, Hcy can be transsulfurated to Cys by two sequential vitamin B_6_-dependent reactions [11]. Besides being an Hcy byproduct, Cys is a GSH precursor inside cells. In turn, the action of gamma-glutamyltransferase (GGT) on extracellular GSH produces glutamate and Cys-Gly, which is usually taken within intracellular milieu by membrane dipeptidases to form Cys and Gly as precursors for GSH resynthesis. Therefore, the major function of GGT activity is to assist in antioxidant defense [10].

Both high plasma Hcy and Cys and low GSH levels are well-known conditions associated with atherosclerosis and CVD [8, 12–14]. More recently, elevated plasma Cys concentrations have been suggested to have a causal role in metabolic diseases, namely, in obesity, insulin resistance, and diabetes mellitus [15, 16].

Plasma aminothiols content and redox state are determined by both genetic and nutritional factors. These last include the intake of sulfur amino acids (Cys and Met), the availability of B-vitamins involved in their metabolism, and the presence of dietary antioxidants (such as vitamins A, C, and E) to inhibit oxidative processes and thereby contribute to a more reduced redox state. Also lifestyle changes, such as tobacco cessation, the practice of physical exercise, and moderate consumption of alcohol contribute for a normal aminothiol profile [17].

Taking into account that the Azores, an Atlantic, Portuguese, volcanic origin archipelago formed by nine islands has a higher mortality rate for CAD when compared to mainland Portugal, we hypothesized that some CVD risk factors could be highlighted in Azorean subjects. Therefore, the aim of this study was to compare the prevalence of standard, as well as some emerging CVD risk factors, namely, the plasma aminothiol profile (and its major determinants) between two groups of healthy subjects from Ponta Delgada (PDL) and Lisbon cities, respectively.

## 2. Subjects and Methods

### 2.1. Subjects and Study Design

The study groups consisted of 101 volunteers from Ponta Delgada city (in São Miguel island, circa 68,000 inhabitants) and 121 from Lisbon city (in mainland Portugal, 547,773 inhabitants). PDL subjects were recruited in collaboration with Unidade de Saúde de ilha de São Miguel (USISM) and those from Lisbon were anonymized participants of the e_COR study carried out by Instituto Nacional de Saúde Dr Ricardo Jorge (INSA), between 2012 and 2015. All subjects were caucasian, aged 20 to 69 years and gave a written informed consent to participate in this study. The study has been performed in accordance with the ethical standards laid down in the 1964 Declaration of Helsinki and its latter amendments.

Participants were requested to answer a questionnaire on their medical history, lifestyle information (physical activity and alcohol and tobacco consumption), and medicine intake. Subjects with a history of CVD, diabetes mellitus, or other chronic diseases, as well as those who were taking vitamins supplements, were excluded from the study.

This study had a cross-sectional design where subjects were tested for standard CVD risk factors and for plasma aminothiol content and its main circulating determinants (plasma methionine, folate, vitamins B_12_, and B_6_ and GGT activity).

### 2.2. Blood Collection

A single overnight 12 h fasting venous blood sample was obtained from all subjects. The blood was drawn into 4.9 ml vacutainer tubes with heparin or into 10 ml tubes without anticoagulant. After centrifugation at 2,500 × g for 15 min at 4°C, plasma or serum were separated and divided into 200 *μ*l aliquots, freshly used or stored at −80°C until HPLC analysis, as adequate.

### 2.3. Definition of Covariates (Risk Factors)

Body weight and height were measured to calculate body mass index (BMI) and waist circumference. Global obesity was defined by a BMI ≥ 30 kg/m^2^. Central (or abdominal) obesity was defined according to the NCEP/ATP III criteria [18] by a waist circumference greater than 102 cm and 88 cm for men and women, respectively. Smoking habits were considered when subjects currently smoked or had stopped smoking in the last year. Alcohol consumers were defined as alcohol intake >0 g/day versus abstainers (=0 g/day). The international physical activity questionnaire (IPAQ) was used to assess practitioners of physical activity (moderate plus active) and nonpractitioners [19]. Hypertension was diagnosed according to JNC-7 criteria [20] as blood pressure (BP) higher than 140/90 mmHg and/or the current use of antihypertensive drugs. The homeostasis model assessment of insulin resistance (HOMA-IR) was calculated as follows: fasting insulin (*μ*U/mL) × fasting glucose (mmol/L)/22.5 and used as a measure of insulin sensitivity [21]. Subjects with HOMA-IR ≥ 2.73 (4^th^ quartile) were considered as insulin resistant. Dyslipidemia was defined according to the NCEP/ATP III [18] criteria by high LDL cholesterol levels (LDL-c ≥ 160 mg/dL) and/or high triglycerides levels (TG ≥ 150 mg/dL) and/or low HDL cholesterol levels (HDL-c ≤ 40 mg/dL) and/or by the use of lipid-lowering drugs. Hyperhomocysteinemia (HHcy) was defined as levels of plasma homocysteine ≥ 15 *μ*M and hypercysteinemia (HCys) as plasma Cys > 300 *μ*M [8]. For vitamin B deficiencies, the following usually considered cutoff points were employed: serum folate < 3 ng/mL; vitamin B_12_ < 200 pg/mL; and vitamin B_6_ < 7.4 ng/mL.

### 2.4. Biochemical Measurements

Blood parameters, such as serum urea, uric acid, creatinine, glucose, GGT activity, and lipid profile, were determined in fresh samples by validated standard laboratory methods. Total (i.e., the sum of reduced, oxidized, and free and protein-bound species) plasma concentrations of Hcy, Cys, Cys-Gly, and GSH were measured by a RP-HPLC methodology with fluorescence detection (Exc. at 385 nm and Em. at 515 nm) and using a platinum EPS C18 analytical column (53 mm × 7 mm I.D., 3 *μ*m particle size), according to the method described in Ferin et al. [22] and already applied in Lima et al. [23]. The chromatographic analysis of the aminothiols was performed by using 0.1 M KH_2_PO_4_ (pH 2.0) containing 40 mL·L^−1^ of ACN as the mobile phase, which allowed well-resolved peaks of the individual compounds. The temperature of the column oven was maintained at 35°C that shows the best resolution and shorter RT. The flow rate of 1 mL·min^−1^ was adopted in our study, which allowed increased heights of the peaks and decreased their respective width and RT. An aliquot of 20 *μ*L was injected through a Rheodyne injection valve after filtration through a 0.45 *μ*m PVDF filter. The identification of four peaks was done by comparison of the RT with those corresponding to the pure standards run separately in the same analytical conditions and confirmed by spike of the authentic standards to the plasma sample.

Plasma Met level was also determined by a RP-HPLC method with fluorescence detection [24]. Serum insulin, folate, and vitamin B_12_ concentrations were analyzed by chemiluminescent microparticle immunoassay on an Architect 2000 analyzer (Abbott Laboratories, USA). Plasma pyridoxal-5′-phosphate level (the active circulating form of vitamin B_6_) was determined by RP-HPLC with fluorescence detection according to the method of Kimura et al. [25]. Serum retinol (vitamin A) and *α*-tocopherol (vitamin E) concentrations were determined by an isocratic RP-HPLC-UV method [26].

### 2.5. Sample Size Determination

Sample size was calculated *a posteriori* to be 23 subjects in each of the two study groups (*N* = 46), by using software G^*∗*^Power (University of Dusselldorf, Germany, version 3.1.9.2, http://www.gpower.hhu.de/en.html), thus validating the final sample size used in this work. The parameters used for calculation were a mean difference in plasma Cys of 50 *μ*M between groups (assuming a standard deviation of also 50 *μ*M) at the 5% level of significance with 90% power.

### 2.6. Statistical Analysis

Results are expressed as mean ± standard deviation (SD) or frequencies as appropriate. Variables were tested for normal distribution by using the Kolmogorov–Smirnov test. Continuous variables were compared by using the Student's *t*-test if they were normally distributed or the Mann–Whitney *U* test otherwise. Categorical variables were compared by using the Fisher exact test or the chi-square test as appropriate. Correlations between selected pairs of variables were evaluated by using Spearman's rank correlation coefficient. To examine the relationship among plasma Cys level or waist circumference and other variables, linear regression analyses were performed. Only the significant variables found at univariate were considered for multivariate analysis. All analyses were performed by using SPSS version 22.0 for Windows (SPSS Inc., Chicago, IL). A 2-tailed *P* value <0.05 was considered significant.

## 3. Results

### 3.1. Baseline Characterization of the Study Groups

Demographic, clinical, and biochemical characteristics of the study groups are described in Table 1. Both groups were matched to age, gender, and BMI (*P* > 0.05). No differences were observed between the groups regarding the prevalence of conventional cardiovascular risk factors, such as global obesity, menopause, smoking, and alcohol consumption, as well as hypertension and dyslipidemia. However, men from PDL revealed to have both higher waist circumference/central obesity and a more sedentary life compared to men from Lisbon. PDL subjects had lower diastolic BP and higher levels of creatinine, glucose, and HOMA-IR score compared to their counterparts from Lisbon, although the latter parameter did not reach statistical significance when analyzed by gender. There were no differences in serum mean levels of urea, uric acid, and insulin, as well as in lipid profile between the study groups, except in HDL-c concentrations. In fact, women from PDL had significantly higher HDL-c concentrations compared to women from Lisbon, where oral contraceptives users were less numerous. PDL subjects (namely, the feminine gender) were also taking more antiaggregant medication, but no differences between groups were registered concerning statins and antihypertensive drugs consumption.

### 3.2. Plasma Aminothiol Profile and Its Determinants in the Study Groups

Table 2 shows the circulating concentrations of aminothiols and their major determinants in the study groups. In addition to individual thiol values, it also includes their substrate/product ratios as indicators of the enzymatic activities where they are involved: Hcy/Cys (cystathionine *β*-synthase and cystathionine *γ*-lyase), GSH/Cys-Gly (*γ*-glutamyl transferase), Cys/GSH (*γ*-glutamyl-cysteine synthetase or glutathione synthetase), and Met/Cys (relative to the transsulfuration pathway).

PDL subjects exhibited higher plasma levels of Cys, Hcy, and Cys-Gly but similar GSH concentration compared to those from Lisbon. It should be noted that plasma Cys was the aminothiol exhibiting by far the highest average concentration difference between the two groups (about 28%, *P* < 0.001). Therefore, a clear higher prevalence of hypercysteinemia was registered in PDL, namely, in male gender, as compared to Lisbon group. Although Hcy mean level was higher in PDL (namely, in feminine gender) than in Lisbon subjects, no differences in the prevalence of hyperhomocysteinemia between the study groups were observed.

Significant lower Hcy/Cys and Met/Cys ratios were found in PDL subjects, when compared to those from Lisbon. However, no difference in GSH/Cys-Gly ratio (an indirect measure of GGT activity) was registered between the study groups. Contrariwise, Cys/GSH ratio was significantly higher in PDL vs. Lisbon group.

The plasma concentration of Met, a major determinant of plasma Cys level, did not differ between the study groups, and it only correlated with vitamin B_12_ levels and tobacco use (*r* = 0.17; *P*=0.013, *r* = −0.18; *P*=0.009, respectively). In fact, PDL subjects exhibited less serum vitamin B_12_ and more folate levels compared to those from Lisbon, namely, in feminine and male gender, respectively. Also, PDL subjects revealed a higher frequency of vitamin B_12_ deficiency compared to their counterparts. Diversely, plasma vitamin B_6_ levels were similar in both groups.

No difference in GGT activity was observed between the male groups, which is in accordance with their similar GSH/Cys-Gly ratios. However, as compared to Lisbon, GGT activity was significantly increased in PDL women, where GSH would be expected to be decreased owing to the strong inverse association observed between the two parameters (*r* = −0.49, *P* < 0.001). Furthermore, GSH was the only plasma aminothiol that was increased in women taking oral contraceptives (2.28 ± 0.8 in users vs. 1.83 ± 0.6 *μ*M in nonusers, *P*=0.007). Nevertheless, when removing oral contraceptive users from analyses, there remained no difference in GSH level between the two feminine groups (1.87 ± 0.5 vs. 1.82 ± 0.6 *μ*M, *P*=0.730). Also, GGT activity did not differ between oral contraceptive users and non-users.

### 3.3. Serum Antioxidant Fat-Soluble Vitamin Concentrations in the Study Groups

Both serum *α*-tocopherol and retinol concentrations were significantly lower in PDL than in Lisbon group (8 ± 1 mg/L in PDL vs. 9 ± 1 mg/L in Lisbon; 0.62 ± 0.2 mg/L in PDL vs 0.66 ± 0.2 mg/L in Lisbon; respectively), but vitamin A did not differ among women (0.61 ± 0.2 mg/L in PDL vs. 0.61 ± 0.2 mg/L in Lisbon). No differences were found in these vitamin concentrations between nonusers vs. users of oral contraceptives.

### 3.4. Correlation Analysis

As expected, all plasma aminothiols correlated with each other, except GSH, which only correlated with Cys-Gly. Plasma Cys (but not Hcy) was positively associated with age (*r* = 0.23; *P* = 0.001, *r* = 0.11; *P* = 0.114, respectively), while GSH was negatively correlated with age (*r* = −0.21, *P* = 0.001). Unsurprisingly, male gender was associated with higher plasma levels of both Hcy and Cys-Gly (*r* = 0.32; *P* < 0.001, *r* = 0.19; *P* < 0.01, respectively), but a similar relationship concerning Cys was only found in PDL group (*r* = 0.25; *P* < 0.05). No relationship between plasma GSH and gender was registered regardless the use of oral contraceptives. Furthermore, plasma Cys levels correlated with BMI (*r* = 0.22; *P* = 0.001), waist circumference (*r* = 0.31; *P* < 0.001), menopause (*r* = 0.23; *P* = 0.010), systolic blood pressure (*r* = 0.25; *P* < 0.001), creatinine (*r* = 0.22; *P* = 0.001), uric acid (*r* = 0.22; *P* = 0.001), glucose (*r* = 0.29; *P* < 0.001), insulin (*r* = 0.22; *P* = 0.001), HOMA-IR (*r* = 0.26; *P* < 0.001), folate (*r* = 0.24; *P* < 0.001), and GGT (*r* = 0.28; *P* < 0.001), as well as with vitamin E (*r* = −0.17; *P* = 0.013). In turn, plasma Hcy levels were associated with smoking (*r* = 0.17; *P* = 0.013), creatinine (*r* = 0.43; *P* < 0.001), uric acid (*r* = 0.40; *P* < 0.001), folate (*r* = −0.30; *P* < 0.001), vitamin B_12_ (*r* = −0.30; *P* < 0.001), and GGT (*r* = 0.22; *P* < 0.001). On the other hand, the plasma Hcy level did correlate neither with obesity parameters nor with those respecting insulin resistance.

### 3.5. Regression Analysis

**Figure 1:**
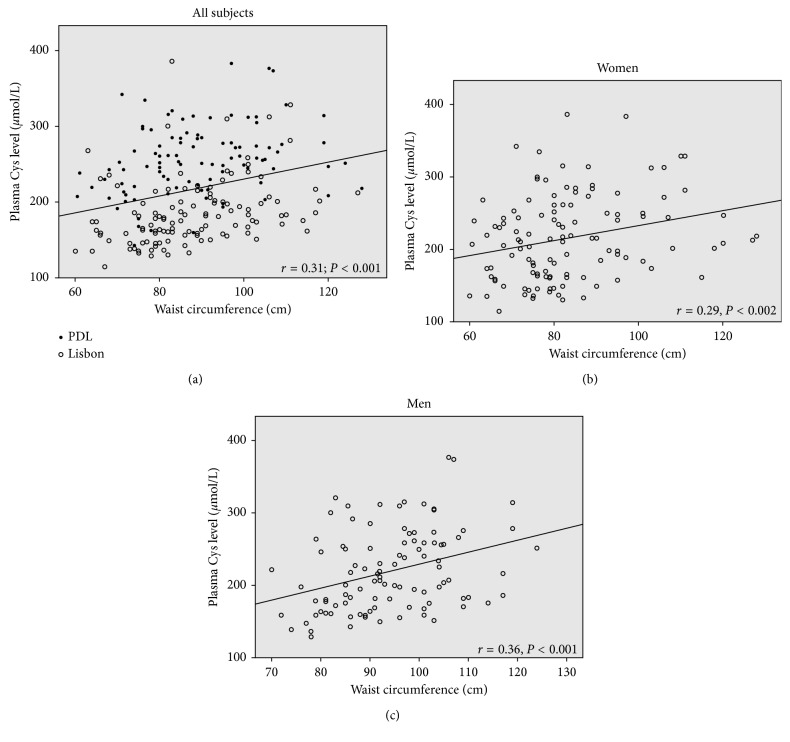
Correlations between plasma Cys level and waist circumference in all subjects from the study groups (a) and by gender (b, c).

In order to evaluate the plasma Cys concentration predictors in all subjects from the two study groups, regression analyses were performed. In univariate regression analyses, age, BMI, waist circumference, menopause, creatinine, glucose, insulin, systolic BP, folate, and GGT were associated with Cys levels. However, when using a multivariable regression model, only creatinine and folate concentrations were independently associated with plasma Cys levels (Table 3). BMI and menopause status were not included in this model owing to their high collinearity with waist circumference and age, respectively. However, when performing the same model to test BMI instead of waist circumference, no prediction on plasma Cys level was found. Regression analysis was also applied to test whether plasma Cys predicted waist circumference (Table 3). In fact, plasma Cys level was found to predict central obesity but not BMI (data not shown). Additional adjustment for Cys confounders (creatinine and folate) increased the significance (*P*=0.010) of Cys predictive value in waist circumference.  Figure 1 shows the bivariate positive correlation between plasma Cys and waist circumference in the study groups.

## 4. Discussion

### 4.1. Conventional Risk Factors in the Study Groups

In this study, PDL and Lisbon groups were age and gender matched and exhibited a similar prevalence of the conventional cardiovascular risk factors (dyslipidemia, hypertension, global obesity, menopausal state, and smoking). This is in accordance with Cymbron et al., [27], who already had reported that the Azores archipelago and mainland Portugal had a similar frequency of risk alleles for dyslipidemia or hypertension. However, in the present work, men from PDL revealed to have a more sedentary life with a concomitant higher waist circumference (central obesity) compared to those from Lisbon.

Surprisingly, women from PDL had higher HDL-c levels compared to those from Lisbon. However, it is known that the use of oral contraceptives (due to the estrogen component) increases serum HDL cholesterol [28]. In fact, oral contraceptives acted as confounders in PDL feminine gender, by having possibly conferred upon them a better antioxidant and anti-inflammatory status.

### 4.2. Hypercysteinemia in PDL Subjects

The simultaneous determination of the four aminothiols levels in plasma has emerged as a useful tool not only for clinical research involving oxidative stress and metabolic and redox regulation but also for monitoring the disease status [22].

PDL subjects have shown a worse pro-oxidant aminothiol profile compared to those from Lisbon, as reflected in their elevated plasma Cys, Hcy, and Cys-Gly concentrations. When present in high levels, these three aminothiols (each one measured as the sum of oxidized and reduced forms) can have pro-oxidant effects [7, 8], through a mechanism involving auto-oxidation and oxidative damage. In fact, their highly reactive thiol (-SH) group is readily oxidized to form ROS, like superoxide anion radical (O_2_^−·^), and H_2_O_2_ in the vascular wall. In turn, excess of O_2_^−·^ can lead to the formation of hydroxyl radical (HO^·^) or react with NO to form peroxynitrite radical, causing a decrease in NO bioavailability and consequent endothelial disfunction and vascular disease [29]. All those reactive species initiate lipid peroxidation and may induce the oxidation of LDL and therefore atherosclerosis and CVD events, as previously referred. The mean plasma Cys levels found in PDL subjects (namely, in male gender) were in the high borderline of the reference interval and similar to those reported by Ӧkzan et al. [12] and Yardim-Akaydin et al. [30] in CAD patients of older age.

#### 4.2.1. High Ingestion of Sulfur-Rich Amino Acids

A high ingestion of sources of sulfur-containing amino acids (Cys plus Met) like animal protein products (red meat, fish, poultry, eggs, milk, and dairy products) in PDL could be partly responsible for these results. In fact, high animal protein diets, which are rich in Met, have already been associated with high plasma Hcy and Cys levels [31, 32] and with increased CAD risk [33, 34]. However, when comparing plasma Met content in the study groups, a slightly lower value was observed in PDL subjects, yet not significant. A possible reason for this is that blood has been collected under fasting conditions and Met in PDL subjects is being overoxidized through the transsulfuration pathway and is not being preserved by the remethylation one, a situation that has been reported to occur when there is a high intake of Met [35]. This hypothesis corroborates the lower Hcy/Cys and Met/Cys ratios found in PDL vs. Lisbon subjects.

Furthermore, no differences were found in plasma vitamin B_6_ levels (an essential dietary cofactor in the transsulfuration pathway) between PDL and Lisbon groups, which reinforces the presumed high bioavailability of animal protein in the diet of PDL subjects.

High-protein diets increase urea levels. However, urea concentrations did not differ between the study groups, which points to a similar protein intake. Nevertheless, results suggest that dietary protein of PDL subjects is richer in sulfur amino acids than that of Lisbon group.

A high ingestion of Met in PDL subjects is also indicated by their elevated serum creatinine concentration when compared to Lisbon, which points out to a higher bioavailability of S-adenosylmethionine (SAM) for creatine synthesis and creatinine formation in PDL. In fact, dietary protein consumption (namely, red meat intake) increases serum creatinine level through protein catabolism [36, 37]. Furthermore, men have higher muscle mass compared to women, turning them more prone to have higher creatinine levels (and a consequent altered aminothiol profile), as shown in the present work (Table 1). Future studies should evaluate SAM levels (as a better index of Met intake compared to plasma Met) in both populations.

#### 4.2.2. Low Availability of Vitamin B_12_

PDL subjects also have exhibited both lower serum vitamin B_12_ levels and much higher vitamin B_12_ deficiency prevalence compared to those from Lisbon. Low vitamin B_12_ levels, even within reference range, have been reported to cause, *per se*, high concentrations of folate (5-methyl-tetrahydrofolate), by diminishing its conversion to tetrahydrofolate and leading to an inevitable reduction of the Hcy remethylation cycle. Admitting, as previously described, a higher animal protein content in the diet of PDL vs. Lisbon subjects, lower levels of vitamin B_12_ (with animal protein origin) are suggested to come from a malabsorption process rather than from an inadequate intake. In addition, low vitamin B_12_ [38] and high folate levels [39] have already been associated to a polymorphism in methionine synthase (MTR c.2756 *A* > *G*), the enzyme catalyzing the folate-dependent remethylation pathway of Hcy [40], which has not been investigated in the present work.

In any case, a low availability of vitamin B_12_ and the consequent folate “trapping,” together with a diet rich in sulfur amino acids (as reflected in creatinine levels) seem likely to be responsible for the hypercysteinemia observed in PDL subjects (Table 3).

### 4.3. Hypercysteinemia and Antioxidant Defenses

The transsulfuration pathway is also redox regulated (at the level of cystathionine *β*-synthase) and is upregulated in situations of oxidative stress, thus favoring GSH synthesis [41]. However, this was not observed in this study, where plasma GSH levels were similar in both study groups. Moreover, there was no correlation between Cys and GSH, which puts forward that plasma Cys (but not Cys-Gly) concentrations in excess did not arise from GSH degradation. Also, Cys/GSH ratio was increased in PDL, indicating that plasma Cys concentration exceeded its requirement for GSH synthesis. Another study also supports that an excess of Cys in diet does not increase plasma GSH concentrations [42] but can inhibit its synthesis instead [43]. Therefore, the similar GSH levels found in PDL and Lisbon groups could reflect an overconsumption of the tripeptide in oxidative stress conditions or a decreased GSH synthesis in PDL subjects, both affecting negatively their antioxidant defense resources.

Serum GGT activity is regarded as an early marker of antioxidant inadequacy, even within the reference range generally accepted [44, 45]. In this study, PDL women have shown a higher GGT activity compared to those from Lisbon, which was correlated with all aminothiols, mainly GSH. This last is a well-known relationship, since the main action of GGT is to metabolize extracellular GSH to produce Cys and Cys-Gly, thus being the main regulator of their circulating concentrations [46]. Besides the higher GGT activity, as well as plasma Cys, Hcy, and Cys-Gly contents in women from PDL vs. Lisbon, plasma GSH levels were similar in both groups, regardless the use of oral contraceptives, which corroborates their similar GSH/Cys-Gly ratios. This suggests that GGT activity was mainly increased in PDL women for some other reason rather than GSH degradation, such as the use of antiaggregant therapy (Table 1).

Regarding male gender, GGT activity (as well as GSH/Cys-Gly ratio) did not differ between the study groups, which reinforces the conclusion that this parameter was not a good early marker of oxidative stress conditions in the present study. In fact, GGT activity would be expected to be elevated in men from PDL, owing to their increased pro-oxidant profile (as evidenced by high levels of plasma Cys, Hcy, and Cys-Gly). In turn, it is clear that the high Cys levels in plasma did not arise from GSH degradation (as reflected in GGT activity), as shown in regression analyses.

### 4.4. Hypercysteinemia: A Causal Factor for Central Obesity in Azores?

According to the present results, our hypothesis is that circulating Cys levels (when in high concentrations) together with a limited physical activity can generate central obesity, which is hastened by proinflammatory and pro-oxidant processes derived from HCys. In turn, central obesity can account for insulin resistance, high blood pressure, dyslipidemia, and further oxidative stress conditions, thus favoring atherogenesis. In this study, as well as in others [15, 47–49], plasma Cys is associated with both global and central obesity and insulin resistance. Several underlying mechanisms are believed to support a causal role of Cys in obesity. First, high Cys levels have been associated with decreased energy expenditure via increased *Scd-1* (stearoyl-CoA desaturase-1) expression and concomitant higher SCD-1 activity, a key lipid synthesizing enzyme and checkpoint in obesity development [50]. Second, Cys can have pro-oxidant effects, as a result of its auto-oxidation (in a Cu^2+^ dependent manner) in plasma by releasing H_2_O_2_, which, through redox cell signaling pathways, inhibits lipolysis and favors lipogenesis; oppositely, decreased GSH levels accelerate adipogenesis, since the tripeptide is needed for glutathione peroxidase activity to scavenge H_2_O_2_ [51]. Third, Cys has insulin-like actions on adipocytes [51], which favors gene expression of lipogenic and diabetogenic enzymes [42].

Interestingly, in the current study, plasma Cys levels predicted central obesity (waist circumference) but not global obesity (BMI). Also, several studies have shown the better predictability of waist circumference over BMI in assessing the risk of cardiovascular risk factors [52–54]. In fact, visceral adipose tissue is more metabolically active and insulin-resistant than subcutaneous adipose tissue. Women, namely, before menopause, have a more peripheral subcutaneous distribution of fat (“pear-shaped”) but menopause induces a more visceral (“apple-shaped”) distribution of fat, similar to male gender [55]. These sex differences were well visible in our study, where plasma Cys was a better predictor of central obesity in men compared to women of similar age (Figure 1). In turn, central obesity is a major risk factor to several diseases, including metabolic syndrome, diabetes mellitus, and CVD [53, 54, 56].

A recent study reported that the Azores archipelago has the highest (global and central) obesity prevalence in Portugal [57]. Also, official statistical data [58] show that feminine gender in Azores is the most obese in the country, supporting our hypothesis of an obesogenic role of hypercysteinemia in Azorean populations.

### 4.5. Hypercysteinemia and Insulin Resistance

High plasma Cys (but not Met) has also been reported to be linked to obesity-related disorders such as metabolic syndrome components [32, 48], diabetes mellitus [16], and CVD [8]. On the other hand, it is well-known that risk toward development of diabetes mellitus is high in obese persons. In the present study, PDL subjects exhibited higher HOMA-IR score as compared to the Lisbon ones. This suggests that high plasma Cys (or central obesity) could also play a role in insulin sensitivity and related disorders in these subjects, where the relationship between plasma fasting glucose and Cys levels was very significant as well (*r* = 0.29; *P* < 0.001). In conditions of excessive Cys levels (as in PDL subjects), this amino acid is degraded to pyruvate, which has the ability to increase glucose levels, through gluconeogenesis, as usually seen in obesity and insulin resistance situations.

As is the case of other cross-sectional studies, a cause-effect association between plasma Cys and central obesity cannot be derived from the present study. However, there is substantial evidence in literature that Cys is a causal factor for obesity and not the reverse [49, 59].

Furthermore, in this study, as shown by regression analyses, Cys was a determinant of central obesity but central obesity was not a plasma Cys determinant, which corroborates the hypothesis that elevated Cys concentration is a cause and not a consequence of obesity.

### 4.6. Limitations of This Study

This work had several limitations. First, about 40% of PDL women were under taking oral contraceptives to prevent pregnancy, which was shown to be a big confounder in some analyses (HDL-c and GSH levels) and possibly has conferred some protection regarding the development of central obesity. Second, it was not possible to determine the methionine synthase activity (suspicious to be decreased in PDL group) and the respective polymorphism in these subjects. Third, unfortunately, data regarding dietary intake were not available to corroborate our results. However, it has recently been proved that higher dietary sulfur-containing amino acids are associated with higher prevalence of obesity, insulin resistance, and inflammation [60].

## 5. Conclusions

In conclusion, the prevalence of either standard risk factors for atherosclerosis or hyperhomocysteinemia is similar in both study groups. However, PDL subjects (namely, men) seem to be more prone than those from Lisbon to develop a pro-oxidant aminothiol profile due to a marked hypercysteinemia, a condition which has been referred to have a causal role in CVD. This seems to arise from different behavioral and lifestyle factors, namely, an unhealthy diet (suggested to be highly rich in sulfur amino acids but poor in fat-soluble antioxidant vitamins) and also a low physical activity in PDL group. Because diet and lifestyle can also influence CAD risk, efforts should be done concerning those items in order to improve CAD primary prevention in the Azores population.

Assuming that hypercysteinemia is a causal factor for obesity, PDL subjects (both genders) will be at a potentially higher risk to suffer from both that condition and related disorders, namely, insulin resistance and CAD, compared to those from Lisbon. Therefore, lowering plasma Cys content (by reducing the ingestion of sulfur-rich amino acids sources and raising the bioavailability of vitamin B_12_) is recommended as a preventive strategy to decrease the prevalence of those diseases in the Azores archipelago.

Data generated from this study allow to partially interpret the higher CAD standardized mortality rate found in Azores archipelago as compared to mainland. Moreover, it shows that the plasma aminothiol profile can be an easily evaluated, precocious, and reliable biomarker of both oxidative stress and related disorders in healthy populations.

## Figures and Tables

**Table 1:** Demographic, clinical, and biochemical characteristics of subjects from Ponta Delgada (PDL) and Lisbon groups.

	All		Women		Men	
	PDL (101)	Lisbon (121)	PDL (61)	Lisbon (59)	PDL (40)	Lisbon (62)
Age (years)	42 ± 13	43 ± 15	42 ± 13	42 ± 16	41 ± 11	44 ± 15
Male (%)	40	51	—	—	—	—
BMI (kg/m^2^)	26 ± 4	26 ± 6	25 ± 5	26 ± 7	27 ± 3	26 ± 4
Obesity (%)	14	17	11	19	17	16
Central obesity (%)	35^*∗*^	21	31	27	40	16^‡^
Waist circumference (cm)	89 ± 15	87 ± 14	84 ± 14	83 ± 15	97 ± 11^‡^	92 ± 11
Menopause (%)	33	36	33	36	—	—
Current smoker (%)	27	26	26	20	27	32
Current alcohol use (%)	70	66	62	47	81	84
No physical activity (%)	43^*∗*^	28	34	27	56^‡^	28
Blood pressure (mmHg)						
Systolic BP	121 ± 17	121 ± 21	120 ± 19	116 ± 22	122 ± 12	126 ± 9
Diastolic BP	73 ± 12^*∗*^	78 ± 12	72 ± 13^†^	77 ± 13	74 ± 9^‡^	80 ± 11
Hypertension (%)	30	30	32	31	27	29
Creatinine (mg/dL)	0.83 ± 0.2^*∗*^	0.78 ± 0.2	0.74 ± 0.1^†^	0.66 ± 0.1	0.97 ± 0.1^‡^	0.91 ± 0.1
Urea (mg/dL)	31 ± 8	32 ± 9	29 ± 8	29 ± 8	34 ± 7	34 ± 8
Uric acid (mg/dL)	5.2 ± 1	5.2 ± 1	4.5 ± 1	4.3 ± 1	6.3 ± 1	6.1 ± 1
Glucose (mg/dL)	90 ± 9^*∗*^	87 ± 11	88 ± 9^†^	84 ± 10	93 ± 8^‡^	89 ± 12
Insulin (*μ*U/mL)	10.3 ± 6	9.2 ± 5	9.9 ± 6	8.7 ± 4	10.9 ± 5	9.6 ± 6
HOMA-IR score	2.4 ± 2^*∗*^	2.0 ± 1	2.3 ± 2	1.9 ± 1	2.5 ± 1	2.2 ± 2
HOMA-IR ≥ 2.73 (%)	32	21	26	17	40	24
Serum lipids (mg/dL)						
TC	197 ± 34	193 ± 38	203 ± 34	197 ± 43	187 ± 32	190 ± 33
HDL-c	60 ± 19^*∗*^	55 ± 14	68 ± 18^†^	61 ± 12	48 ± 13	49 ± 14
LDL-c	117 ± 29	121 ± 35	116 ± 30	119 ± 40	119 ± 29	124 ± 29
TG	105 ± 57	105 ± 54	99 ± 54	100 ± 49	113 ± 60	110 ± 58
Dyslipidemia (%)	37	41	28	34	50	47
Medicine intake (%)						
Oral contraceptives	39^*∗*^	14	39^†^	14	—	—
Statins	16	15	13	17	20	13
Antihypertensives	23	20	23	24	23	16
Antiaggregants	12^*∗*^	3	13^†^	0	10	5

Values are presented as mean ± SD, except otherwise indicated. Figures in parentheses are the number of subjects (*n*). ^*∗*^*P* < 0.05 for Lisbon group vs. PDL group; ^†^*P* < 0.05 for Lisbon women vs. PDL women; ^‡^*P* < 0.05 for Lisbon men vs. PDL men.

**Table 2:** Plasma aminothiol profile and its major determinants in Ponta Delgada (PDL) and Lisbon groups, according to gender.

	All		Women		Men	
	PDL (101)	Lisbon (121)	PDL (61)	Lisbon (59)	PDL (40)	Lisbon (62)
Total plasma aminothiols						
Cys (*μ*M)	255 ± 47^*∗*^	185 ± 44	247 ± 47^†^	182 ± 52	269 ± 43^‡^	188 ± 36
HCys (%)	18^*∗*^	4	13	5	25	3^‡^
Hcy (*μ*M)	11 ± 3^*∗*^	10 ± 5	10 ± 3^†^	8 ± 3	12 ± 4^‡^	11 ± 5
HHcy (%)	12	10	8	3	17	16
Cys-Gly (*μ*M)	30 ± 6^*∗*^	27 ± 6	29 ± 6^†^	26 ± 6	32 ± 6^‡^	28 ± 5
GSH (*μ*M)	1.99 ± 0.6	1.88 ± 0.7	1.97 ± 0.6	1.93 ± 0.8	2.03 ± 0.6	1.83 ± 0.6

Substrate/product ratios						
Hcy/Cys	0.04 ± 0.01^*∗*^	0.05 ± 0.03	0.04 ± 0.01^†^	0.05 ± 0.02	0.05 ± 0.01^‡^	0.06 ± 0.03
GSH/Cys-Gly	0.07 ± 0.02	0.07 ± 0.03	0.07 ± 0.02	0.08 ± 0.03	0.06 ± 0.02	0.07 ± 0.03
Cys/GSH	140 ± 53^*∗*^	112 ± 48	138 ± 56^†^	106 ± 43	144 ± 48^‡^	118 ± 52
Met/Cys	0.06 ± 0.02^*∗*^	0.10 ± 0.04	0.07 ± 0.02^†^	0.10 ± 0.05	0.06 ± 0.02^‡^	0.10 ± 0.04

Aminothiol determinants						
Met (*μ*M)	16 ± 5	18 ± 7	16 ± 6	18 ± 7	16 ± 4	19 ± 7
Folate (ng/mL)	9 ± 3^*∗*^	8 ± 3	9 ± 3	9 ± 4	9 ± 3^‡^	7 ± 3
Deficiency (%)	0	3	0	2	0	5
Vit. B_12_ (pg/mL)	364 ± 160^*∗*^	445 ± 168	349 ± 179^†^	449 ± 189	386 ± 125	440 ± 146
Deficiency (%)	19^*∗*^	6	21^†^	5	15	7
Vit. B_6_ (ng/mL)	17 ± 10	17 ± 12	15 ± 9	15 ± 5	21 ± 11	19 ± 16
Deficiency (%)	6	2	8	3	3	0
GGT activity (U/L)	26 ± 21^*∗*^	20 ± 15	21 ± 17^†^	14 ± 6	34 ± 23	27 ± 19

Values are presented as mean ± SD, except otherwise indicated. Figures in parentheses are the number of subjects (*n*). HCys, hypercysteinemia; HHcy, hyperhomocysteinemia. ^*∗*^*P* < 0.05 for Lisbon group vs. PDL group; ^†^*P* < 0.05 for Lisbon women vs. PDL women; ^‡^*P* < 0.05 for Lisbon men vs. PDL men.

**Table 3:** Variables with potential influence on plasma Cys levels or waist circumference in all subjects from PDL and Lisbon groups, tested by multiple linear regression analysis.

Variables	Standardized *β*-coefficient	*P* value
*Plasma Cys*		
Age	0.021	0.805
Waist circumference	0.165	0.062
Systolic BP	0.049	0.541
Creatinine	0.182	0.009
Glucose	0.045	0.543
Insulin	0.014	0.863
Folate	0.301	0.001
GGT	0.031	0.669
*Waist circumference*		
Male	0.226	0.001
Age	0.131	0.042
Hypertension	0.138	0.016
Urea	0.046	0.397
Uric acid	0.139	0.036
HOMA-IR	0.376	0.001
Dyslipidemia	0.136	0.011
Cys	0.102	0.034
GGT	0.031	0.570

## Data Availability

The data used to support the findings of this study are available from the corresponding author upon request.
